# Intravenous rehydration of malnourished children with acute gastroenteritis and severe dehydration: A systematic review

**DOI:** 10.12688/wellcomeopenres.12346.1

**Published:** 2017-08-18

**Authors:** Kirsty A. Houston, Jack G. Gibb, Kathryn Maitland

**Affiliations:** 1 Department of Paediatrics, Faculty of Medicine, Imperial College, London, W2 1PG, UK; 2 KEMRI-Wellcome Trust Research Programme, Kilifi, 80108, Kenya

**Keywords:** malnutrition, gastroenteritis, dehydration, rehydration, systematic review, Africa, Asia

## Abstract

**Background: **Rehydration strategies in children with severe acute malnutrition (SAM) and severe dehydration are extremely cautious. The World Health Organization (WHO) SAM guidelines advise strongly against intravenous fluids unless the child is shocked or severely dehydrated and unable to tolerate oral fluids. Otherwise, guidelines recommend oral or nasogastric rehydration using low sodium oral rehydration solutions. There is limited evidence to support these recommendations.

**Methods:** We conducted a systematic review of randomised controlled trials (RCTs) and observational studies on 15 ^th ^June 2017 comparing different strategies of rehydration therapy in children with acute gastroenteritis and severe dehydration, specifically relating to intravenous rehydration, using standard search terms. Two authors assessed papers for inclusion. The primary endpoint was evidence of fluid overload.

**Results:** Four studies were identified, all published in English, including 883 children, all of which were conducted in low resource settings. Two were randomised controlled trials and two observational cohort studies, one incorporated assessment of myocardial and haemodynamic function. There was no evidence of fluid overload or other fluid-related adverse events, including children managed on more liberal rehydration protocols. Mortality was high overall, and particularly in children with shock managed on WHO recommendations (day-28 mortality 82%). There was no difference in safety outcomes when different rates of intravenous rehydration were compared.

**Conclusions:** The current ‘strong recommendations’ for conservative rehydration of children with SAM are not based on emerging evidence. We found no clinical trials providing a direct assessment of the current WHO guidelines, and those that were available suggested that these children have a high mortality and remain fluid depleted on current therapy. Recent studies have reported no evidence of fluid overload or heart failure with more liberal rehydration regimens. Clinical trials are urgently required to inform guidelines on routes and rates of intravenous rehydration therapy for dehydration in children with SAM.

## Abbreviations

AGE, acute gastroenteritis; BNP, B type natriuretic peptide; CI, confidence interval; ED, Emergency Department; FEAST, Fluid As A Supportive Therapy; GEMS, Global Enteric Multi-Centre Study; HSD/D5, Half Strength Darrow’s and 5% dextrose; HR, Heart Rate; IV, Intravenous; ORS, oral rehydration solution; RCT, randomised controlled trial; ReSoMal, Rehydration Solution for Malnutrition; RL, Ringers Lactate; SAM, severe acute malnutrition; WHO, World Health Organization.

## Introduction

Severe acute malnutrition (SAM) is directly responsible for over 500,000 child deaths per year and plays a significant contributing factor in the deaths of many more ^1^. The World Health Organization (WHO) recommends ten key steps for inpatient management of complicated SAM, and suggest that strict adherence to this protocol should reduce mortality to less than 10% ^2, 3^. With regard to cases complicated by diarrhoea, the 2003 WHO Guidelines for the inpatient treatment of severely malnourished children states that ‘Dehydration, usually resulting from profuse watery diarrhoea (3 or more per day), is often difficult to diagnose in malnourished children because the clinical signs usually relied on to diagnose dehydration are similar to those found in severe wasting without dehydration ^2^.’ Nevertheless, a prospective study of unselected Kenyan children hospitalised with SAM published in 2012 showed that diarrhoea (defined as three or more watery stools) complicated 49% of cases at admissions to hospital and developed in another 16% following admission with case fatalities of 21% and 18% respectively compared with 12% in cases without diarrhoea (χ ^2^ = 17.6 p<0.001) ^4, 5^. Furthermore, whilst the guidelines indicate that signs of severe dehydration (sunken eyes and decreased skin turgor) in SAM are unreliable markers of hydration status (and challenges arise with recognition of dehydration in children with kwashiorkor) the Kilifi study showed that signs of severe dehydration were more common in those with diarrhoea (35% versus 8%) and was a risk factor for mortality (crude odds ratio 1.7; 95% confidence interval (CI) 1.1, 2.6; p = 0.012). In the multivariate model, bacteraemia (odds ratio 6.7 (95% CI 2.5-17.8 p<0.001) and hyponatraemia (odds ratio 4.9 (95% CI 2.2-11.1 p<0.001) were key risk factors for mortality ^5^.

Fluids are the mainstay of treatment for dehydration; however, WHO rehydration strategies in severely malnourished children are extremely cautious. The guidelines advise oral or nasogastric rehydration using ReSoMal (rehydration solution for malnutrition, a hypo-osmolar solution with lower sodium and higher levels of potassium than standard oral rehydration solution) and strongly recommends against the use of intravenous fluids unless the child is shocked. For the shocked child (or child with severe dehydration who is unable to tolerate oral fluids), WHO guidelines recommend 15mls/Kg of intravenous fluid over 1 hour (repeated once if necessary), and a 10ml/Kg blood transfusion over 3 hours if there is no subsequent improvement ( Table 1) ^3, 6^. These are based on concerns regarding susceptibility to fluid overload in children with malnutrition and concerns regarding the use of additional sodium. In the section of the 2003 guidelines (under Step 4: Correct Underlying Electrolyte Imbalance/), the recommendations state ‘All severely malnourished children have excess body sodium even though plasma sodium may be low ( *giving high sodium loads will kill’*) ^2^. There is no published physiological evidence to support this contention.

**Table 1.** WHO recommendations for treatment of severely malnourished children with dehydration[2].

	Shock * and severe dehydration in child unable to tolerate oral fluid	No shock
Initial	15ml/Kg Ringers Lactate + 5% dextrose **OR** ½ Strength Darrow’s + 5% dextrose, over 1 hour, repeated once if needed If no improvement: Transfusion 10ml/Kg over 3hours (start 4ml/Kg/hour maintenance while awaiting blood)	ReSoMal ^#^ Oral/Nasogastric – 5ml/kg every 30 minutes for first 2 hours
Subsequent	Oral/Nasogastric ReSoMal alternating with F75 10ml/Kg/hr up to 10hrs, and then refeeding with F75.	Then 5–10ml/kg/hr alternating F75 ^$^ and ReSoMal for 4–10 hours

*Shock is defined as presence of all three of the following: prolonged capillary refill time (CRT >3s), temperature gradient and weak and fast pulse ^#^ReSoMal – rehydration solution for malnutrition, ^$^F75 – primary feeding formula for children with SAM

Researchers observing children with SAM have postulated that this group have compromised cardiovascular function and are therefore susceptible to fluid overload. Therefore, clinical practice has avoided aggressive fluid therapy in this group of children.

WHO guidelines referring to management of children with SAM were revisited in 2013 and remain unchanged with no mention of any of emerging new data ^6^. The recommendations continue to be based on expert opinion (strong recommendations based on weak level of evidence) rather than clinical evidence. Therefore, we have conducted an independent systematic review of the current available evidence underlying use of intravenous rehydration for children with dehydration and SAM.

### Objectives

To conduct a critical appraisal of available evidence on the safety of intravenous (IV) rehydration therapy for treatment of severe dehydration in children with SAM.

## Methods

We did not publish a protocol prior to conducting this review. A search of online literature was performed. There were pre-determined criteria, as detailed below for eligibility of studies, data outcomes, and an assessment of risk of bias and study method quality in each of the identified studies. English search terms were used.

### Selection criteria

***Population.*** Children aged 0 to 12 years with SAM who had received IV fluids for management of severe dehydration secondary to gastroenteritis or shock. We used the WHO definitions for malnutrition (weight for height (WHZ) <-3, mid-upper arm circumference (MUAC) <115mm or oedema consistent with kwashiorkor), gastroenteritis (three or more loose watery stools per day) and for severe dehydration (presence of two of the following signs: reduced skin turgor, sunken eyes, inability to drink, lethargy or reduced consciousness) ^3^. We excluded studies with chronic or persistent diarrhoea lasting ≥ 14 days.

***Intervention and comparison.*** All studies that evaluated intravenous fluids were included with a specific focus on safety. Studies were excluded if they considered rehydration in children without malnutrition, or only considered rehydration via the oral or nasogastric route.

***Outcome.*** Clinical studies that reported on any outcomes were included. The primary outcomes for this review were incidence of fluid overload following fluid administration (defined as clinical or echocardiographic evidence of heart failure or pulmonary oedema or the need for diuretics), as an indicator for safety, and urine output in response to rehydration therapy as a marker for efficacy. Secondary outcomes of interest were all-cause mortality, and other assessments of IV fluid safety and frequency of other fluid related adverse events: neurological compromise (defined as any reported seizure activity, altered consciousness or unequal pupils); cardiovascular compromise (defined as any worsening of blood pressure, bradycardia or tachycardia); or/and development or worsening of hyponatraemia (since WHO recommends hypo-osmolar intravenous fluids for resuscitation).

***Study design.*** Randomised-controlled trials (RCTs) and observational studies were included.

### Search methods

***Online database search.*** A comprehensive literature search of the following databases was conducted on the 15 ^th^ June 2017 using the terms ‘fluid’ AND ‘malnutrition’ AND ‘children’ AND ‘rehydration OR dehydration’:

- PubMed/ Medline
- Global Health Library (Virtual Health Library)
- Cochrane Database of Systematic Reviews
- Cochrane Central Register of Controlled Trials
- ClinicalTrials.gov
- The WHO International Clinical Trials Registry Portal (ICTRP) search portal

Each of the eligible studies was assessed and a manual review of the reference lists carried out. Additionally, a Google search was performed. The search was limited to trials published in English language.

**Figure 1.**
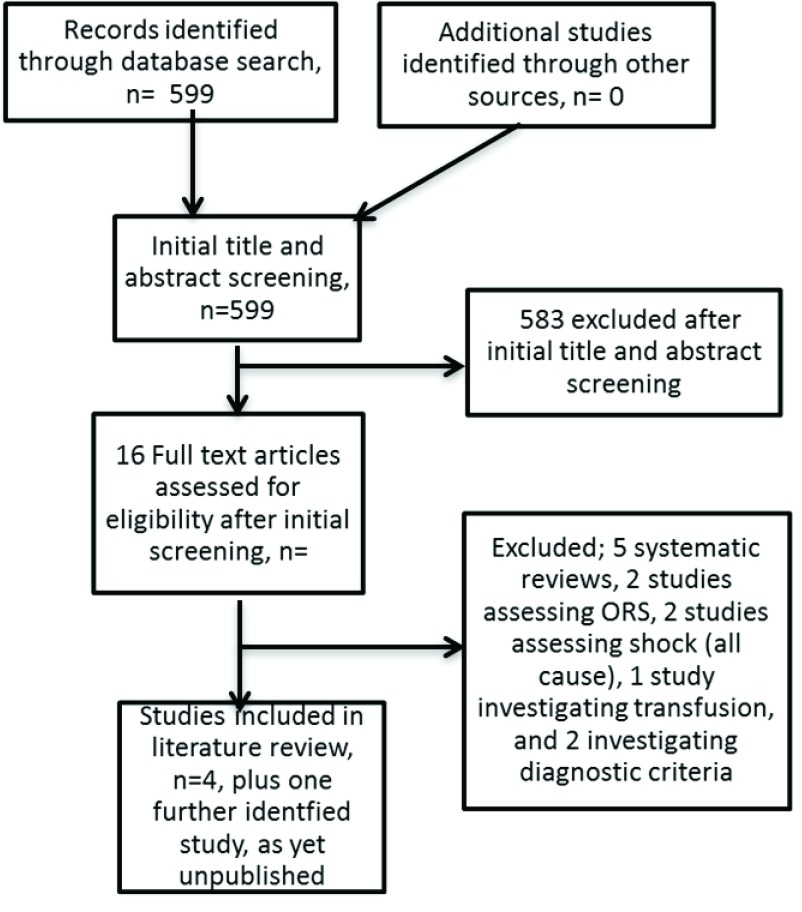
Flow diagram for selection of studies and reasons for study exclusion.

The authors screened the results of the literature search for studies that met the inclusion criteria as determined by the PICOS outline (see Figure 1).

## Results

### Study selection

The search produced 599 studies (see Figure 1.) After screening and evaluation, four studies were identified that investigate use of intravenous fluid treatment in children with severe malnutrition complicated by dehydration, incorporating a total of 883 patients. All four of these studies were conducted in low resource settings (Kenya ^7, 8^, Uganda ^8^ and Bangladesh ^9, 10^). Two of these studies were randomised controlled trials ^7, 10^, and two were observational studies ^8, 9^. Two considered different rates of rehydration ^7, 8^ and both incorporated serial assessment of haemodynamic responses, urinary output, with one including sequential detailed echocardiographic and electrocardiographic assessment before and after receipt of fluids. The third study evaluated the use of a standardised protocol for management of dehydration ^9^ and the fourth trial treated all children with severe dehydration with IV fluids (assessing safety) and then randomised children to one of three oral rehydration solutions (ORS) ^10^. One study included children with cholera only ^10^ (see Box 1).

**Box 1.** Management of cholera in children with severe acute malnutrition

**WHO Guidelines** **(2013) ^3^**	The only indication for intravenous infusion in a child with SAM is shock OR a child with severe dehydration and who cannot be rehydrated orally or by nasogastric tube. These children should receive 15ml/Kg/hour of either ½ strength Darrow’s + 5% dextrose OR Ringers lactate +5% dextrose. Children should be monitored every 5–10minutes for signs of over-hydration and congestive heart failure. If there is no improvement, a blood transfusion (10ml/Kg over at least 3hours) should be given.
**Evidence for intravenous rehydration**	This review includes 266 children with cholera out of a possible total of 802 (33%) from two studies that identified children with cholera (Alam *et al.* and Ahmed *et al.*) ^9, 10^. Ahmed did not perform any sub-analyses on children with cholera. Relevant findings from Alam 2009: 149 (85%) children presented with severe dehydration and required IV rehydration (mean amount of IV fluid required was 103 ml/Kg (95%CI 96-109)) No significant difference in baseline electrolyte abnormalities No children died in this study and no children developed signs of fluid overload No child developed signs of hyponatraemia (not specified) All children were clinically rehydrated within 6 hours although 31% did not pass urine within this period Rice-ORS group had significantly less stool output
**Implications in practice**	There is just one study evaluating safety of intravenous rehydration in children with SAM and cholera. This study rehydrated 149 children with a mean amount of 103ml/Kg of ‘cholera saline’ (sodium 133 mmol/L, potassium 13 mmol/L, chloride 98 mmol/L) and did not report any adverse outcome from this treatment i.e. no fluid overload, no significant difference in dysnatraemia, mortality or fluid related adverse effects. Guidelines for cholera in children with SAM remain extremely conservative and are at risk of undertreating children.

There was some heterogeneity in the population eligibility criteria, sample size, and methods employed by each study, and in their results. Table 2 and Table 3 show the setting, methodology and features of the included studies and their results. One further study was identified on clinicaltrials.gov that assessed two rates of IV rehydration in children with SAM. This study was conducted and completed in Bangladesh. The corresponding author was contacted and indicated that the report is currently under review for publication ^11^.

**Table 2.** 

Author	Year	Location	Study type	Population	Sample size	Inclusion	Exclusion	Comparison	Outcomes
**Obonyo *et al.*^8^**	2017	Kilifi, Kenya and Mbale, Uganda	Prospective observational	Children aged 6–60mths	20	Severe malnutrition (any one of; MUAC <11.5cm, WHZ <-3 or oedema indicative of kwashiorkor, with signs of severe dehydration (>3 watery stools/ 24 hours), and shock (two or more of the WHO criteria)	Severe dermatitis of the groin, congenital heart disease, and consent not given	**Group 1:** WHO standard protocol; 15ml/Kg Ringers lactate over 1 hour, with option to repeat once if signs of shock persist, followed by IV ½ strength Darrow’s/5% dextrose given at 4ml/Kg/hour **Group 2:** 10ml/kg/hour of Ringers lactate over 5 hours	Clinical, haemodynamic and echocardiographic data were collected
**Akech *et al.*^7^**	2010	Kilifi District Hospital, Kenya	RCT	Children aged >6mths	61	Severe acute malnutrition (WHZ <-3 or WH% 70% or MUAC <11cm or oedema involving at least both feet (kwashiorkor) with evidence of shock (1 or more of: CRT>2 seconds, lower limb temperature gradient, weak pulse volume, deep acidotic breathing, creatinine >80μmol/L, or depressed conscious state.	Severe anaemia (<5g/dl), pulmonary oedema, raised intra cranial pressure or known congenital heart disease	**Group 1:** Severe dehydration/ shock: randomly assigned to receive either WHO fluid resuscitation regime (HSD/5D 15mls/Kg over 1 hour, then repeat bolus and if no improvement 10mls/Kg blood) **OR** Ringers lactate (10mls/Kg over 30 mins repeated only twice over 1 hour if features of shock remained **Group 2:** Presumptive septic shock: randomly assigned to either WHO fluid resuscitation regime OR Ringers lactate OR 5% Human Albumin solution	Primary outcome was resolution of features of shock at 8 and 24 hours. Secondary outcomes included incidence of adverse events and mortality
**Alam *et al.*^10^**	2009	ICDDR, B Dhaka Hospital, Bangladesh	RCT	Children aged 6 to 60 mths	175	Severe malnutrition (WHZ <70% of NCHS mean, Acute watery diarrhoea of <48hrs duration, presence of V. cholera under microscopy	Dysentery, severe infections	Randomly assigned to receive one of 3 formulations of ORS with same salt composition, but different substrates i.e. glucose, glucose plus ARS and rice powder.	Primary outcome was stool output Secondary outcomes was time to oedema free weight for length of 80%
**Ahmed *et al.*^9^**	1999	ICDDR,B Dhaka Hospital, Bangladesh	Observational Cohort study	Children aged 0 to 5 years	627	Severely malnourished children, admitted with diarrhoea, whose WFH or WFA was less than 70% and 60% of NCHS median, respectively, or who had oedema	None specified	Non-protocol group i.e. children admitted between Jan 1, 1996 and June 30, 1996 (rehydration usually over 3–6hours) and standardised- protocol group (Jan 1, 1997 and June 30, 1997). Standardised protocol included slow rehydration (if severe dehydration given 20ml/Kg for 1 hour then 10ml/Kg for 1 hour, ORS was started after 1 hour) with an emphasis on oral rehydration, immediate feeding of defined diet, routine vitamins/micronutrient supplements, broad spectrum antibiotics	Mortality rate Others included transfers to nutritional units, and discharge rate

MUAC Mid upper arm circumference, WHZ Weight-for-height Z score, CRT Capillary refill time, NCHS US National Centre for Health Statistics, ICDDR, B International Centre for diarrhoeal disease research, Bangladesh

**Table 3.** 

	Risk of bias	Methodology	Evidence of fluid overload or cardiac failure and urine output	other Outcomes (Fluid related adverse events and efficacy of treatment)
**Obonyo *et al.*,** **2017 ^8^**	Low	Observation of children following WHO standard protocol (Group 1) was halted after 10 patients were enrolled and investigators were concerned about high mortality rate so 10 patients were prospectively recruited to received 10ml/Kg/hour over 5 hours i.e. without bolus treatment. All children were switched to ReSoMal once able to tolerate oral or NGT fluids. Blood transfusion if Hb <5g/dl. And all other treatments received as per WHO guidelines for severe acute malnutrition. Serial clinical assessments, echocardiographs and ECG’s.	None of the children developed echocardiographic or clinical features of over-hydration or cardiac failure. Satisfactory urine output (>1ml/Kg/hr) in 8/11 (73%) in group 1 and 7/9 (78%) in group 2	Mortality at 48hours and Day 28 was reported Group 1: 35% (4 deaths) and 81.8% (9 deaths) respectively Group 2: 44% (4 deaths) and 55.6% (5 deaths) respectively This difference was not statistically significant None of the patients classified as having WHO shock survived Group 1: 4 early deaths (within <24 hours) were all due to cardiovascular collapse secondary to hypovolaemia. 1 child died at 48 hours due to cardiovascular collapse. 4 late deaths occurred (48 hours to 15 days) due to aspiration of feeds and two dying in the community. Group 2: 3 early deaths due to cardiovascular collapse and 1 due to respiratory arrest (severe acidosis and hypoxaemia). Two late deaths occurred at day 4 (1 respiratory arrest associated with metabolic complications and 1 in the community) All fatal events were judged as unrelated to fluid challenges or the IV fluid regime, and largely were secondary to underlying comorbidities Bradycardia at admission (10% of patients) and low systolic blood pressure and persistent weak pulse were associated with early death, the majority within 48hr of admission.
**Akech *et al.*,** **2010 ^7^**	Low	Randomised 1:1 in strata i.e. severe dehydration/shock and presumed septic shock. Unblinded trial: fluids received fluid as per randomisation. Continuous haemodynamic and clinical monitoring. Urine output measured. Blood and urine sample series taken. In all other aspects children were treated as per WHO SAM guidelines	No children developed clinical features of pulmonary oedema or allergic reaction during the course of study observation Oliguria at 8 hours was present in 9/22 (41%) in HSD/5D arm vs. 3/25 (12%) in RL arm, p=0.0	Overall 31/61 (51% died): No significant difference in mortality between arms. 26/31 (84%) fulfilled WHO malnutrition shock definition at admission. High case fatality in this group irrespective of allocated intervention 39% (12/31) deaths occurred within 24hours of recruitment and 52% within 48hrs) No differences in mean sodium concentration at admission, 8 and 24hours between arms. Resolution of shock: at 8 and 24hours proportion of children with shock in arms was considerable, but not significantly different between groups
**Alam *et al.*,** **2009 ^10^**	Low	Randomised 1:1:1. Fluid as per randomisation. Blinding of clinicians and patients. Children with severe dehydration received intravenous ‘cholera saline until recovered from shock or severe dehydration. Estimated fluid deficit was corrected with assigned ORS. Same ORS solution given to match stool losses and purging losses. Children with some dehydration were randomised to receive assigned ORS within 1 hour, and those with severe dehydration within 6hours of admission after IV rehydration. Serial blood samples, stool and urine taken.	None of the children had or developed clinical evidence of cardiac failure or fluid overload 31% of all children (including those receiving ORS only) remained oliguric at 6 hours, and 12% at 12 hours	All of the children were clinical rehydrated within 6hours and 31% did not pass urine within this period however by 12 hours 88% of the children produced urine and by 24hours all had produced urine. Hypernatraemia or severe hyponatraemia and severe hypo or hyperkalaemia was not observed in any child Groups statistically differed in stool output and in ORS and water intakes. The difference was entirely contributed by rice-ORS group children who had the greatest reduction in stool output and the ORS volume taken was less in the rice-ORS group and water intake greater. There was no statistical difference in duration of cholera. None of the children died in this study
**Ahmed *et al.*,** **1999 ^9^**	Moderate	Comparison of cohorts receiving two protocols. Non-protocol i.e. prior to implementation of standardised protocol, received intravenous fluid over 3 to 6 hours if severely dehydration. Antibiotics only if clinically indicated. Feeding delayed until rehydration completed. Micronutrients not given routinely. Standardised protocol had slower IV fluids and emphasis on oral rehydration, standard antibiotics and micronutrients and standardised management of hypoglycaemia and hypothermia.	No evidence of fluid overload or cardiac failure was reported in this study Urine output not reported	Twice as many children on non-protocol treatment developed hypoglycaemia during hospital stay (6% vs. 3%) 199 (59.5%) of children in standardised protocol group were successfully rehydrated with oral rehydration rather than IV fluids, compared with 85 (29%) in the other group (p<0.0001) Total volume of IV fluids was smaller and duration shorter in standardised protocol group (p<0.0001) More children in non-protocol group became critically ill and required special care treatment or needed treatment with ceftriaxone. More children on standardised protocol were discharged uneventfully 73% vs. 63%, p=0.006. 30 (9%) mortalities in standardised protocol versus 49 (17%) in non- protocol group (odds ratio=0.49 (95% CI 0.2-0.8), p=0.003) Children who died on standardised protocol died mostly within first 48 hours. Younger age, poorer nutritional status, increased frequency of hypoglycaemia, bacteraemia and greater volume of IV fluids infused were risk factors for death

NGT Nasogastric tube, ECG electrocardiogram, ORS Oral rehydration solution, ReSoMal Rehydration solution for malnutrition, WHO World Health Organization, SAM Severe acute malnutrition

### Risk of bias

The quality of each of the included studies was assessed for risk of bias using the Cochrane collaboration’s tool in order to evaluate validity ^12^. Three of the studies had a low overall risk of bias ^7, 8, 10^, while the remaining study had a moderate risk due to limitations in the methodology ^9^. 

### Outcomes

***Primary outcome***

*Incidence of fluid overload*

This outcome was available from three studies ^7, 8, 10^. No evidence of fluid overload was found in any of these studies. Alam *et al.* (2009) defined heart failure as tachycardia, tachypnoea, enlarged liver, and prominent neck veins ^11^. Akech *et al.* (2010) reported the need for furosemide or the diagnosis of pulmonary oedema as defined by crepitations in both lungs in the presence of hypoxaemia (low oxygen saturations measured by pulse oximetry) ^7^. Obonyo *et al.* (2017) used a definition of bi-basal crepitations and worsening oxygen saturation (indicative of pulmonary oedema) gallop rhythm, raised jugular venous pressure and increasing hepatomegaly. Obonyo *et al.* (2017) also evaluated myocardial function using echocardiography and electrocardiographic assessment, which did not demonstrate any evidence of myocardial dysfunction indicative of biventricular failure prior to or as a result of intravenous fluid therapy ^8^. They demonstrated that children showed signs consistent with hypovolaemia and myocardial responses to this at baseline and following intravenous fluid therapy resulted in increases in markers of myocardial performance (fluid-responsiveness) in the majority of patients.

*Urine output*

Three studies reported on urine output in response to rehydration therapy. Alam *et al.* measured urine every 6 hours (collected in urine collectors) and reported that 31% of children remained oliguric at 6 hours, and 12% by 12 hours ^10^. However, it should be noted that this included all children, of which 147 had received intravenous fluids for 4 to 6 hours and 26 that received only ORS. Akech *et al.* measured urine output hourly for the first 8 hours (using urinary catheters) and reported frequency of oliguria (<1ml/kg/hour) at 8 hours (which also by the study’s definition included resolution of shock and absence of oliguria). Oliguria was found to be common in both arms, but more in the group receiving half strength Darrow’s and 5% dextrose (9/22, 41%) than in those receiving Ringers lactate (3/25, 12%), p=0.05 ^7^. Obonyo *et al.* measured urine output using urinary catheters and reported ‘satisfactory urine output’ (>1ml/Kg/hour) in 8/11 (73%) of children group 1 and 7/9 (78%) children in group 2 ^8^.

***Secondary outcomes***

*Mortality*

All of the studies reported on mortality rates. One study in Asia reported no deaths in 175 severely malnourished children included in the trial ^10^, whilst the other three reported mortality rates ranging from 9% to 81.8%. Obonyo *et al.* reported mortality at 48 hours and 28 days and found a universally high mortality between both groups: 35% and 81.8%, respectively, in Group 1 (receiving WHO management), and 44% and 55.6% respectively in Group 2 (receiving rehydration alone). Of note was that in children fulfilling the WHO shock criteria none survived ^8^. Akech *et al.* ^7^ also found a high overall mortality (31/61, 51%) with no statistically significant difference between the two arms in a Phase II trial of 61 children; 39% of these deaths occurred in the first 24 hours and 52% within 48 hours. Ahmed *et al.* reported 30 (9%) case fatalities in the standardised protocol ^9^, in which children received IV rehydration over a longer duration i.e. more than 3–6 hours, and use of IV fluids was avoided where possible (children also received a full package of care including IV antibiotics for a minimum of 48 hours, vitamin A prophylaxis, and standardised management for hypoglycaemia) versus 49 (17%) in non-protocol group, in which children received fluid as per WHO non-SAM guidelines; 75ml/Kg oral or nasogastric fluid over 4 hours for children with ‘some’ dehydration and 100ml/Kg IV fluid as per Plan ‘C’, i.e. over 3 hours in children >1 year and over 6 hours if <1 year for those with severe dehydration (odds ratio=0.49 (95% CI 0.2-0.8), p=0.003). Children in the standardised protocol mostly died within the first 48 hours following admission. Young age, poorer nutritional status, increased frequency of hypoglycaemia, bacteraemia and greater volume of IV fluids infused were all risk factors for death ^9^.

There was significant between-study heterogeneity in mortality rates. Mortality was greatest when signs of shock were observed ^8^. There was no statistical difference reported when different rates of IV rehydration were adopted.

*Hyponatraemia*

The definitions of hyponatraemia varied across the studies, so summary data could not be generated. Alam *et al.* (2009) reported an overall baseline rate of hyponatraemia (sodium <130mmol/L) in 38/175 (22%), with no cases of severe hyponatraemia (serum sodium<115mmol/L) at baseline. No child developed features of severe hyponatraemia at any time during the study (features observed were not specified and post fluid sodium level was not recorded) ^10^. Obonyo *et al.* (2017) found that 4/11 (36%) and 5/9 (63%) patients in Groups 1 and 2 respectively were found to have severe hyponatraemia (serum sodium <125mmol/L) at baseline ^8^. Akech *et al.* (2010) found 4/26 (15%) and 3/29 (10%) cases of hyponatraemia (<125mmols/L) at baseline in the half strength Darrow’s and 5% (HSD/D5) and Ringers lactate (RL) groups, respectively. They also demonstrated mean plasma sodium increases from baseline to 24 hours of 133 to 138mmol/L in the HSD/D5, and 134 to 140mmol/L in the RL group ^7^. Data on the post-fluid rates of hyponatraemia were not published in Alam *et al.* or Ahmed *et al.* studies ^9, 10^, although common at baseline.

*Neurological complications*

No studies reported any incidences of seizures. However, it is unclear if this outcome was predefined for inclusion in any of these studies.

*Cardiovascular compromise*

Two studies undertook detailed cardiovascular evaluations. Akech *et al.* (2010) found that, overall 19/55 (34%), 10/47 (21%) and 12/39 (30%) children were severely tachycardic (heart rate>160 beats/minute) at baseline, 8 and 24 hours after starting fluid respectively. Significantly more were tachycardic at 24 hours in the group receiving HSD/D5 group than the RL group (44% vs. 16%, p=0.04). No incidences of bradycardia (HR<60 beats/minute) were reported ^7^. Obonyo *et al.* (2017) published mean age adjusted heart rate and systolic and diastolic blood pressures of survivors and fatalities at baseline, and between 30 minutes and then 8 hour intervals up to 48 hours. They found that a low systolic blood pressure and a persistent weak pulse after IV fluid administration were associated with death in 11/20 (55%) patients. Longitudinal monitoring demonstrated decreases in heart rate in both groups over the first 48 hours with no significant differences between mortalities and survivors. Diastolic blood pressure was lower in patients in the rehydration only group who died before 4 hours, suggesting uncorrected hypovolaemia. No evidence for biventricular heart failure was found. In this study, children were monitored additionally using cardiac biomarkers (Troponin I, a highly sensitive biochemical marker of myocardial damage, and brain natriuretic peptide (BNP), a marker of volume expansion and pressure loading, which has previously been used to predict outcome in paediatric heart failure ^13– 15^ at admission and 48 hours. Troponin I remained within the normal range in both groups. BNP was elevated (>300pg/ml) in 6/10 (60%) in group-1 and 2/9 (22%) at baseline and remained elevated in 4/7 (57%) patients in Group 1 but not in any patients in Group 2. The interpretation of this is challenging and requires further exploration.

## Discussion

All studies in the review included children with SAM, diarrhoea and some level of dehydration ranging from 41% with any dehydration ^9^ to 100% with severe dehydration ^8^. Two focused on shock (since this is the only indication for use of fluid therapy recommended by WHO), whilst two studies extended the management to children without strict WHO shock or rehydration regimes that are currently recommended by WHO in order to generate pilot data on safety and preliminary data on outcomes (efficacy) ^7, 8^. Critical to the evidence underpinning current treatment recommendations and contrary to the accepted opinion that the malnourished heart is susceptible to failure if intravenous fluids are administered, none of the studies demonstrated any evidence of fluid overload in their respective cohorts. These findings highlight the lack of available data to support current guidelines recommending conservative fluid therapy in rehydration of children with SAM and pave the way for further research to inform future practice.

Concerns regarding use of intravenous fluids in this vulnerable group of children reference impaired cardiovascular function and susceptibility to overload. A number of studies have been conducted over the last 30 years in Africa, Asia, the Americas and Europe that evaluate cardiovascular function in children with SAM using Echocardiography ^16– 25^. However, it should be noted that none of these additional studies have been conducted in the context of the child with SAM receiving rehydration therapy, with the exception of the study by Obonyo *et al.* ^8^. We summarise the findings below.

A number of early studies described reduced cardiac mass, decreased left ventricular function and cardiac and stroke indices in malnourished children when compared with healthy children. Viart *et al.* (1977) measured clinical and haemodynamic parameters in 43 Jamaican children with marasmic kwashiorkor and compared them with 24 convalescent children. In the malnourished children, haemodynamic parameters were abnormal when compared with convalescent patients: red cell volume and total blood volumes were 51% and 66%; cardiac and stroke indices averaged 58% and 62%, of the convalescent values, respectively. Malnourished children therefore showed prolonged circulation time with associated bradycardia and hypotension when compared with convalescent children ^23^. Similarly, two observational studies (Singh *et al.*, 1989, Shoukry *et al.*, 1986) conducted in India included 63 children with malnutrition, and found that malnourished children had smaller cardiac mass. In particular, left ventricular mass was less and indicators of left ventricular function were reduced (including cardiac output and stroke volume) ^18, 19^. *Bergman et al* (1988) observed 21 children with kwashiorkor in South Africa and also found that they had low cardiac dimensions ^17^. Phornphatkul *et al.* (1994) monitored cardiovascular status before, during and after nutritional rehabilitation and found that Thai children with SAM had features indicative of impaired ventricular function, as shown by change in fractional shortening (p= 0.015), mean velocity of circumferential fibre shortening (p= 0.038), and systolic time interval (p= 0.030) ^24^. More recently, Olivares (2005) compared 30 malnourished and 30 healthy Spanish children and found that left ventricular mass and left ventricular index was significantly lower in children with malnutrition (left ventricular mass: 55.3 ± 10.3 *vs.* 71.4 ± 6.9 g, *p* = 0.000; left ventricular mass index: 46.5 ± 6.6 *vs.* 60.5 ± 4.9 g/m2, *p* = 0.000) ^20^. However, these studies are constrained by their small sample sizes and none considered indexing the heart muscle in relation to general muscle mass.

Contradicting the findings of these earlier studies, four studies assessing cardiovascular function in children with SAM conducted between 1992 and 2016 found that, in SAM, there appears to be relative ‘cardiac- sparing’. Kothari *et al.* (1992) found that cardiac mass is reduced compared with that of well-nourished children; however cardiac weight: total body weight ratio is higher (4.44 +/- 1.45 vs. 2.42 +/- 0.87; p<0.001, 95% C.I. 1.28 to 2.76), and systolic function was not significantly different to well-nourished children. Cardiac output was therefore considered as appropriately reduced in proportion with body size ^22^. Ocal *et al.* (2001) found that left ventricular mass was lower in malnourished children but in proportion to body surface area, and therefore cardiac indices were not significantly different from normal ranges ^21^. El-Sayed *et al.* (2006) agreed that cardiac mass index was significantly lower in children with malnutrition, but that left ventricular function was not significantly reduced ^16^. The largest and most recent study (Silverman *et al.* 2016) evaluating baseline cardiovascular function in 272 ‘stable’ Malawian children demonstrated no significant differences in cardiac index, stroke volume index and heart rate between inpatient children with and without SAM ^25^.

As evidenced by the four studies included in this systematic review, there has been no reported fluid overload in children treated with IV fluid when dehydrated. To the contrary, Obonyo *et al.* (2017) found that ‘despite a high mortality rate, neither clinical nor echocardiographic data indicated evidence of volume overload’. There was also no evidence of gross myocardial dysfunction by echocardiographic or by specific cardiac biomarkers including Troponin I and BNP. Fluid administration in both groups led to improvements in stroke volume index and left ventricular fractional shortening, and Obonyo *et al.* concluded that ‘further research is required to investigate whether more liberal fluid rehydration strategies may be beneficial’ ^8^.

Finally, the cardiac myocardial physiological study in children with SAM (CAPMAL study) conducted in Kilifi County Hospital between May 2011 and February 2012 included 88 SAM cases (52 with marasmus and 36 with kwashiorkor phenotypes) and 22 non-malnourished disease-matched controls. Serial echocardiographic and electrocardiographic data were gathered over the course of admission and additional measurements were conducted on any SAM child requiring intravenous fluids. Overall, there was no difference in myocardial function (indexed to body surface area) in cases with either marasmus or kwashiorkor; and no differences between cases and disease matched controls (matched for comorbidities such as pneumonia, diarrhoea). Fifteen episodes of fluid resuscitation were recorded involving 12 SAM cases (75% marasmus), the majority (10/11) for hypovolaemia secondary to diarrhoea early in the course of admission. No clinical evidence of a deterioration, indicative of pulmonary oedema, was witnessed. Echocardiographic indices suggested an adequate physiological response to fluid resuscitation ^26^.

This body of evidence undermines the physiological rationale of fluid overload secondary to myocardial dysfunction as a mechanism of death during rehydration in paediatric SAM, and lends support to the notion that this group of children are likely to remain under-filled when following current guidelines. The most compelling data suggesting superiority of one strategy over another with regards to mortality come from Ahmed *et al.* (1999) ^9^. However, combining multiple therapies into a package of care confounds the determination of the causative intervention for improved mortality. Additionally, the pre- and post-intervention design precipitates significant performance bias. Neither Obonyo *et al.* (2017) nor Akech *et al.* (2010) were powered to detect a difference in mortality between the intervention arms. However, the high overall mortality in these studies can be explained by the high prevalence of children with shock at admission ^7, 8^.

There is no compelling evidence to support the conservative approach to rehydration across the spectrum of disease encompassed by the four studies identified in this systematic review. Fluid overload as a consequence of IV fluids in SAM complicated by dehydration has not been detected.

### Application of guidelines

In practice, the WHO guidelines are complex, challenging to follow and open to wide interpretation (see Table 1) (3); the clinician must decide whether to rehydrate the child over 4 or 10 hours or any interval between the two limits, and at what point the child is eligible for intravenous rather than oral rehydration. Additionally, the feasibility of alternating F75 and ReSoMal every 30 minutes in a busy, pressurised and low resource setting is limited ^6^.

For children with shock, the fluid regimen is restricted to a maximum of two slow IV infusions over a total of two hours, with advice to transfuse if no improvement. The recommendation of a transfusion to manage hypovolaemic shock in the context of severe dehydration is extremely concerning as it will not replete the volume (water and electrolytes) loss. As detailed above, the evidence for this level of caution with IV fluid administration is limited and may risk persistent hypovolaemia and lethal shock. Additionally, there are no compelling data that support the superiority of oral over IV rehydration in non-shocked children with severe dehydration.

Difficulties also arise when the child is near to the threshold for SAM and may be misclassified due to dehydration. Prospective research has found that 20% of children hospitalised with acute gastroenteritis (AGE) and severe dehydration (10% or more loss of body weight) temporarily fulfil anthropometric criteria for SAM (MUAC <11.5cm or WHZ <-3SD), but following rehydration they are reclassified as undernourished ^27^. Thus, the current recommendations have much wider implications, with potentially 20% of children with severe dehydration secondary to AGE and without SAM receiving low volume low sodium rehydration, which may explain the poor outcomes that have been observed in the large case-control study Global Enteric Multicentre study (GEMS). This was conducted in Africa and Asia, and showed that patients with moderate/severe gastroenteritis are 8.5 times more likely to die than non-gastroenteritis controls ^28, 29^. A third of the fatalities occurred < 7 days following hospitalisation - indicating that current management strategies may not be working in practice for children with and without SAM and emphasising the need to revisit the current guidelines.

## Conclusions

The WHO guidelines for rehydration in SAM are not supported by high quality evidence. There is also no evidence to support concerns regarding fluid overload in this population on the contrary, haemodynamic evaluation indicates that these children remain under-filled. In the light of recent famines across Africa and the current cholera outbreaks, we are extremely concerned that children with SAM (and potentially 20% of non-SAM children) are having intravenous rehydration fluids withheld on the grounds of strong recommendations based on low quality of evidence. New evidence on myocardial function and from clinical trials suggests they should be afforded the same standard of care as children without malnutrition. Further research should urgently evaluate safety and efficacy of a more aggressive approach to rehydration and support development of evidence based, user-friendly and directive guidelines.

## Supplementary material

Supplementary File 1: PRISMA checklist.
