# Supplementary material for: Intravenous rehydration of malnourished children with acute gastroenteritis and severe dehydration: A systematic review
Source: Wellcome Open Res. 2017 Aug 18;2:65. [Version 1] doi: 10.12688/wellcomeopenres.12346.1 (PMC5590082; doi:10.12688/wellcomeopenres.12346.1)
Supplement: Peer review history

# Peer review: Referee response for version 1

**DOI:** 10.21956/wellcomeopenres.13368.r25146

**Published:** 2017-10-03

**Authors:** Bhargavi Rao

**Affiliations:** 1 Manson Unit, Médecins Sans Frontières (MSF), London, UK

Rehydration in children with SAM is fraught, extremely difficult in field/emergency conditions and frankly unclear as to best practice. This review is sorely needed, so the conclusion that more research is required was disappointing.

 Despite a large number of studies found through an initial search - only four heterogeneous studies passed through the filters, assessing complications of the use of IV fluids in SAM children.  The studies were a mixture of RCTs and observational, as well as varied in their inclusion criteria, treatment protocols and end points.

 It was difficult as a reviewer to understand the descriptions of the different studies due to this variation.  Overall there appeared to be no association between the rate of fluid resuscitation and signs of fluid overload, although one study (observational - Ahmed *et al*) showed a higher mortality in the high volume groups. However, the pesentation of the studies in the paper makes it a little hard to compare the results effectively.  In addition, most studies identified were small, often with only sub-groups eligible for inclusion, which means that extended analysis is not possible. Only 2 of the 4 studies assessed children who meet the WHO criteria for shock. 

 Although all were in resource-limited contexts, none were in emergency settings (Kenya, Uganda and Bangladesh).  One of the studies from Bangladesh looked at children with cholera, rehydrated at a mean rate of 18-25ml/kg/hr (NB WHO recommendation is 30ml/kg over 2 hours i.e. 15ml/kg/hr approximately).  My understanding is that the pattern of fluid loss in cholera is different from other forms of GI loss and mechanism of dehydration - and it would have been good to see some acknowledgement of this in the discussion section, especially since the mortality outcomes in this paper were better than the others.

 Similarly - it would have been useful for the authors to discuss the possible effect of the different mechanisms by which malnutrition occurs - particularly acute on chronic scenarios.

 However - this is clearly a significant clinical issue with scanty evidence and the possibility of high mortality.  Perhaps the authours could have also searched the grey literature in case of reports from agencies/NGOs etc that share their experience tthrough alternative means.

 Overall - the authors have given us cause for thought.  Even though the sample size is not large enugh to permit extended analysis and the studies are heterogenous - they do suggest that that WHO guidelines are too narrow and cautious.  There is definitely enough evidence presented here to warrant a proper study of the dehydration/shock management in SAM children - and in fact perhaps several studies given the variety of mechanisms of dehydration (e.g. cholera vs other gastroenteritis) as well as degrees of shock.

 I congratulate the authors on identifying an important problem with a very limited evidence base in the face of possibly misguided WHO protocols.

I have read this submission. I believe that I have an appropriate level of expertise to confirm that it is of an acceptable scientific standard.

# Peer review: Referee response for version 1

**DOI:** 10.21956/wellcomeopenres.13368.r25178

**Published:** 2017-09-07

**Authors:** Michael Yung

**Affiliations:** 1 Pediatric Intensive Care Unit, Women's and Children's Hospital, Adelaide, SA, Australia

The aim of this review was to investigate the available evidence for the WHO recommendations of cautious intravenous rehydration in children with severe acute malnutrition and severe dehydration. Caution is advised because of the assumptions that malnourished children have excess body sodium and reduced left ventricular mass and function. The authors found only four heterogeneous studies. There was no evidence for harm from fluid overload or heart failure.

 Reading the descriptions of individual studies was difficult because they differed in design, populations, interventions and outcomes, so I reviewed the original studies. One observational study ^1^ specifically addressed the question of heart failure and fluid resuscitation. The study was small (n=20) and found no association between heart failure and the rate of fluid resuscitation. The second observational (also sequential) study compared up to 30 to 100 ml/kg fluid resuscitation ^2^. There were no patients with heart failure in either group. However it is unclear how this was defined and sought. The higher volume group had higher mortality: 17 vs 9% with volume of intravenous fluid an unadjusted risk factor for death. Thus undetected heart failure causing death cannot be excluded. One randomised, controlled trial (RCT) ^3^ comparing two different fluid regimens: isotonic fluid given relatively rapidly and hypotonic fluid (as per WHO recommendations) given relatively slowly. This study did not have fluid overload/cardiac failure as its primary outcome but rather shock resolution. No patients developed clinical signs of pulmonary oedema. As a pilot study it lacked power, and the presence of clinically-apparent pulmonary oedema is a crude sign of cardiac failure, but is clinically relevant and easily detectable. The second RCT ^4^ randomised severely malnourished cholera patients to one of three oral rehydration solutions but intravenous resuscitation was given to all those severely dehydrated (n = 149) at a mean volume of 100 ml/kg. The rate of administration was not stated. The study specifically defined and sought signs of heart failure. No child developed such signs.

 Houston *et al *formulated a specific question, whether intravenous fluid resuscitation in severely malnourished children with diarrhoeal dehydration or shock was associated with heart failure. They used a search strategy including multiple electronic databases and reviewing the reference lists in the resultant studies. However they did not hand search the key, relevant journals, searched the non-English literature or the “grey” literature e.g. conference abstracts, doctoral theses. The inclusion criteria were explicit and risk of bias was assessed. Which author(s) selected and assessed the studies was not stated, neither was how disagreement was resolved.

 That said, the authors have identified an important problem lacking evidence. The little available evidence suggests that a cautious approach to fluid resuscitation in severely malnourished, dehydrated or shocked children may be misguided, denying these children urgently needed intravenous fluids. They correctly conclude that further research in this area is needed urgently.

I have read this submission. I believe that I have an appropriate level of expertise to confirm that it is of an acceptable scientific standard.

## References

[1] ObonyoNBrentBOlupot-OlupotPBoele van HensbroekMKuipersIWongSShiinoKChanJFraserJvan WoenselJBMMaitlandK: Myocardial and haemodynamic responses to two fluid regimens in African children with severe malnutrition and hypovolaemic shock (AFRIM study). *Crit Care*.2017;21(1) : 10.1186/s13054-017-1679-0 103 10.1186/s13054-017-1679-0 28468633PMC5415747

[2] AhmedTAliMUllahMChoudhuryIHaqueMSalamMRabbaniGSuskindRFuchsG: Mortality in severely malnourished children with diarrhoea and use of a standardised management protocol. *The Lancet*.1999;353(9168) : 10.1016/S0140-6736(98)07499-6 1919-1922 10.1016/S0140-6736(98)07499-6 10371570

[3] AkechSOKarisaJNakamyaPBogaMMaitlandK: Phase II trial of isotonic fluid resuscitation in Kenyan children with severe malnutrition and hypovolaemia. *BMC Pediatr*.2010;10: 10.1186/1471-2431-10-71 71 10.1186/1471-2431-10-71 20923577PMC2973932

[4] AlamNHIslamSSattarSMoniraSDesjeuxJF: Safety of rapid intravenous rehydration and comparative efficacy of 3 oral rehydration solutions in the treatment of severely malnourished children with dehydrating cholera. *J Pediatr Gastroenterol Nutr*.2009;48(3) :318-27 1927478810.1097/mpg.0b013e318180af27

# Peer review: Referee response for version 1

**DOI:** 10.21956/wellcomeopenres.13368.r25147

**Published:** 2017-08-24

**Authors:** Elizabeth Molyneux

**Affiliations:** 1 College of Medicine, University of Malawi, Blantyre, Malawi

Severe acute malnutrition (SAM) when compounded by severe gastroenteritis, with or without shock, is daunting to treat. WHO in an effort to simplify treatment has an overall management plan based on several studies of haemodynamic and cardiac function in children with acute SAM. The studies were small, ranging from 21 to 63 enrollees and are now rather old (1977-1994). In those studies of children with severe gastroenteritis, blood volume, cardiac mass, left ventricular function, cardiac output and stroke volume were all impaired in children with SAM when compared with findings in well-nourished children. These results led WHO experts to recommend extreme caution when giving fluids to children with SAM and to avoid IV fluids if at all possible. More recent studies have reported similar findings but have related them to the body surface area or body mass and found them to be proportionately normal.

 Hypovolaemia requires fluid replacement, and if shock is present most emergency paediatricians would want to replace fluids aggressively. This is at odds with WHO advice.

 The FEAST study did not enrol children with either severe malnutrition or gastroenteritus: the caution for fluids in non hypovolaemic shock expressed after that study therefore cannot be expanded to include children with SAM.

 There is also the question of what type of fluid to use for IV administration. WHO recommends half strength Darrow’s solution with 5% dextrose or Ringer’s lactate with 5% on the grounds that children with SAM have a total body sodium greater than normal. These fluids are hypo-osmolar and would not be chosen as replacement fluids for fluid loss or shock in any other emergency situation.

 In this review Houston *et al *searched for relevant articles on the use of IV fluids in children with SAM plus gastroenteritis, with the aim of finding if there were any complications, especially of fluid overload. The literature is sparse and they were able to identify only four articles, two RCTs and two observational trials. Even these four are heterogenous in aims, inclusion criteria, study treatment protocols, observations and end points; but they do all include some children with SAM and severe dehydration with or without shock who received IV fluid administration.

 I found reading about the different studies quite confusing as the studies were so different in design and objectives; I had to go to the original papers for more detail. If table 2 had more details in the column concerning the sample size of each study it would help. Most studies are too small to allow for much analysis and in several studies only a proportion of the children qualify for inclusion in this review. For instance Akech *et al *enrolled 61 children but only 26 had severe dehydration. Different fluid regimens and rates of fluid administration were used, and the aetiologies of diarrhoea varied among studies. Importantly, none of the children developed fluid overload.

 In the study of children with cholera, 147 patients were given a mean of 103 ml of IV fluid over a 4-6 hr period (25-18ml/kg/hr, in contrast to the WHO protocol’s recommendation for SAM with severe diarrhoea of 30ml/kg  maximum in 2 hours.  The mechanism of gastrointestinal fluid loss in cholera is different from mechanisms in other causes of gastroenteritis, and most clinicians would reduce their caution and give extra fluids to this group of patients.  Among the cholera patients in this study, 147 children received IV fluids and there were no deaths. This is very different from the case fatality rates of 9-81% in the other three studies.

 The causes of malnutrition are multiple and complex. Malnutrition is seldom simply due to poor intake except in famine conditions; even then infections both acute (e.g. chest infection) and chronic (e.g. TB, HIV) may compound the problem. Gastroenteritis also differs in cause, acuteness of onset and duration. A difficult clinical scenario is acute on chronic diarrhoea in a severely malnourished, probably septic child.

 Where does this leave us? These studies, though they only include small numbers of children, all suggest that WHO guidelines for how to give IV fluid to a severely dehydrated child with SAM are too cautious. There have been enough pilot studies to justify a large, well run study of the subject. It needs to be large enough to answer questions not only as to how much fluid should be given but also what fluid, at what rate and by which route. The sample size must allow for sub-analyses of SAM children with and without oedema, shock, degrees of anaemia and other confounders.

I have read this submission. I believe that I have an appropriate level of expertise to confirm that it is of an acceptable scientific standard.
